# Model of Intellectual Disability and the Relationship of Attitudes Towards the Sexuality of Persons with an Intellectual Disability

**DOI:** 10.1007/s11195-012-9285-1

**Published:** 2012-11-06

**Authors:** Monika Parchomiuk

**Affiliations:** Faculty of Pedagogy and Psychology, Institute of Pedagogy, University of Maria Curie Sklodowska in Lublin, Narutowicza Street 12, 20-004 Lublin, Poland

**Keywords:** Social model of disability, Individual model of disability, Sexuality, Intellectual disability, Medical students’ attitudes towards sexuality, Poland

## Abstract

The following article discusses the relationship between the model of intellectual disability and the attitudes towards sexuality of people with disabilities. This correlation has been verified during the author’s own research conducted on students of several medical faculties such as nursing, public health, emergency medical services and physiotherapy. Tools of the author’s design have been used in the research. Likert-type scale “Perspective of intellectual disability” has been used to determine the model of disability seen from the medical (individual) or social perspective. To examine the attitudes towards sexuality two tools of the author’s own design have been used: a Likert-type scale “The essence of sexuality in persons with an intellectual disability” which has been used to analyze the cognitive aspect of the attitudes, and a semantic differential with notions concerning physical and psychosocial aspects of sexuality including the affective-evaluative aspect. As expected, significant correlations have been found between the model and the attitudes both in the cognitive and the affective-evaluative aspect. Higher scores for the individual model correlated with: (a) lover scores for most aspects of sexuality of people with intellectual disability, (b) perceiving them as asexual, (c) biological determinism in the sexual sphere. The social model concurred with positive values given to sexuality of people with intellectual disability and its normalization in the sphere of its determinants and symptoms.

## Introduction

To understand social attitudes towards the sexuality of persons with an intellectual disability it is necessary to approach disability itself from a broader perspective. Such a perspective may be offered by the existing models of disability, such as the traditionally recognized individual or social ones. The type of discourse employed in each model reveals how disability is conceptualized, including the causes, manifestations and results, as well as the nature of the postulated types of support, the subjects of this support and forms of providing it. The emergence of models of disability, understood as the creation of certain theoretical perspectives as a basis for analyzing phenomena results from the desire to understand the situations of persons with a disability across time.

Chronologically, the first model of disability to appear was the individual one, also called “biological” or “medical.”^1^ The assumptions characteristic for this model focused on the individual with the disability, and in particular, on his or her biological (physical) defects. The defects that led to limitations in functioning were treated as the basis of the disability. From the perspective of the individual, disability was assumed to be the source of a “personal tragedy.” Professional activities, mostly of a medical nature, focused on adjusting to the state of limited functioning seen in a reductionist way—as accepting the loss (of ability or independence). The key aspect of the individual (medical) model was its reduction or ignorance of the significance of the individual’s activity in the process of coping with the effects of disability, as well as neglecting his or her personal experience. Hence, this model seems limited and to a certain extent deterministic in perceiving the essence of disability [1]. The medical (biological) model, alongside with its basic concept of impairment, treated the sexuality of disabled persons in the categories of a medical problem. Impairments of bodily structures and functions, expected to lead to the inability to satisfy one’s sexual needs, were one of the elements of experiencing the “personal tragedy.” In treating sexuality, the dominant tendency was biologism and medicalization of professional support activities. The medical approach to sexuality saw its essence in the ability to engage in sexual intercourse. An inability to take part in it, due to physical limitations, led to the false generalization regarding the lack of sexual needs of persons with a disability (specialists focused mainly on persons with a physical disability) [2]. The sexuality of individuals with intellectual disability was not evaluated as positive, even from a physical perspective. It was not considered an element of their “personal tragedy” or an object of activity aimed at reaching an adjustment to the disability—as opposed to the case of persons with a physical disability.

The social model emerged as the result of a desire of persons with disabilities to change their own lives towards having more control over it, as well as a greater participation in civic life [1, 3]. In this context, as an ideological contradiction to the individual model, it opposed the assumption about the biological origin of disability, and situated this origin in society. In this perspective, the social model defined disability as the product of specific social and economic structures, and its main interest focused on the problems of oppression and discrimination of disabled persons [4]. However, assumptions of this type had a limited explanatory scope to elucidate the essence (genesis) of disability [4]. With time, those who propagated the social model (especially disabled persons themselves) stressed the significance of cultural aspects such as norms and values that describe socially desired features, behaviors, forms of activity and role fulfillment. As disability is not always the result of material barriers, it may also be a social construct, a result of the assignment of meaning to existing differences between people. This meaning depends on cultural factors, and the same differences may lead to inclusion or exclusion of persons with disabilities. The experience of disability is not so much the result of limitations caused by defects of the body, but of social perception, labeling and certain attitudes towards persons who differ from the physical or mental norm. The aim of professional activities is to reduce physical, economic and social barriers with active participation of the persons with disabilities. The notion of adjusting to disability is broadly understood here—in the personal and social dimension [3].

The social model of disability brought a certain interest in the sexuality of people with disabilities, mainly in the context of related civil rights (the right to marriage, procreation, protection against unconditional sterilization). Activists who represented the social model focused on the external limitations of the sexuality of people with disabilities, and hence, paid attention to ones that were beyond the personal dimension (of impairments and deficiencies). In this context, the social, cultural, ideological and environmental barriers were analyzed [5, 6]. In the social model, the sexuality of people with disabilities escaped from under the “power of specialists” to become an object of reflection concerning personal experiences [6].

The above remarks illustrate that the sexuality of people with disabilities is to be viewed from a broad perspective that considers the essence of disability itself. The literature referring to the above issues includes studies of a theoretical character that follow this approach. The aim of the study was to verify whether a relationship exists between the perception of intellectual disability as an expression of the individual or social model of disability and the attitudes towards the sexuality of persons with intellectual disabilities—in students of medical specializations. The choice of representatives of medical specializations as research participants was motivated by two reasons: (1) the significant role these persons may play in the therapy, education and support of persons with intellectual disabilities, which is part of the professional tasks of the participants; (2) the specific professional preparation that may expose them to the assumptions of the medical model.

The scope of the research on students of medical specializations or on medical professionals to date mainly concerned their general attitudes towards disability, often without differentiating its type [7–9], as well as certain specific aspects of these attitudes, resulting from their professional situations [8, 10]. Also available are studies on the attitudes of medical students towards persons with intellectual disabilities [11–14]. Research on attitudes towards the sexuality of persons with intellectual disabilities, including students of obstetrics as participants [15], nurses employed as caring staff at institutions for disabled persons [16], personnel who work with intellectually disabled persons within the scope of their professional competences (nurses, physiotherapists, general practitioners) of various forms [17–19], addressed several issues within the broadly understood area of sexuality of persons with intellectual disabilities.

The operationalization of the intellectual disability model was based on the proposal by Kirenko [1], which employed the assumptions of the individual (medical) and the social model, created within the program “Human rights and disabled persons” in cooperation with the Polish Sejmik of Disabled Persons. The individual (medical) model of intellectual disability utilized the following conceptual categories: institutionality of the life conditions, dependence on the help of others, biological determinism (impairment of the central nervous system) in the genesis of the phenomenon, the necessity to be subordinate in the process of support, the inability to exercise the rights and duties of a civic type, and medicalization of the professional support activities. The social model was based on: living in an open, natural environment, autonomy, a variety of disability conditions, the ability to make decisions jointly in the process of support, a civic model of life, access to rights and duties, as well as the need for professionals of various fields to cooperate in the support process. In the present study, attitude is understood as a relatively stable tendency in making certain evaluations (mostly positive or negative) of elements and phenomena referring to human sexuality in its biological, mental and social dimension concerning persons with intellectual disabilities.

The specific research aims were to answer the following questions:

1. Which model of intellectual disability—the individual (medical) or the social one—is most frequently endorsed by the participating students?
2. What are the students’ attitudes towards the sexuality of persons with intellectual disabilities, especially regarding the emotional-evaluative and cognitive aspect (beliefs)?
3. Is there a correlation between the medical model of disability and the attitudes towards the sexuality of persons with intellectual disabilities, including the emotional-evaluative and cognitive aspects; and if so, what is its nature?

The research results in this field to date [20, 21] and analysis of the educational standards within the selected specializations of study programs permit the prediction of a stronger occurrence of the medical model among the participants. Based on the above theoretical reflections, a hypothetical solution to the third question from among the formulated research problems may be suggested; namely, that the disability model is related to attitudes towards sexuality. Persons whose attitudes endorse the individual (medical) model are likely to express negative opinions, i.e., ones that focus on deficits and difficulties in the sexual sphere, seeing their origins in the intellectual disability itself, describing the sexual sphere from the perspective of deficits and deviations from the norm, at the same time treating it in an extreme way. In this context, it may also be assumed that the evaluative opinions referring to the sexuality of persons with an intellectual disability are likely to be rather negative. Persons whose attitudes endorse the social model of disability, in turn, are more likely to perceive the sexuality of people with an intellectual disability in a holistic way, i.e., including its biological and psychosocial conditions, recognizing its developmental value and attributing it a status of normality. It is assumed that the emotional-evaluative aspects of their attitude towards the sexuality of persons with an intellectual disability are likely to be rather positive.

## Methods

The Likert type scale, called “The model of an intellectual disability”, was used to determine the model of intellectual disability. It consists of 12 statements, 6 of which refer to the individual model, and 6 to the social one (see Appendix). The participant expresses his or her opinion using a 6-point scale (from 5–I completely agree, to 0–I completely disagree). Cronbach’s alpha coefficient for the whole scale is 0.72. The discriminatory power rating of the questions ranges from 0.21 to 0.54.

The present study on attitudes towards sexuality used two tools unique to this study: a Likert type scale, called “The essence of sexuality of persons with an intellectual disability” (with responses ranging from 5–I completely agree—to 0–I completely disagree, as well as the response X–I am not able to tell) to assess the cognitive dimensions of the attitudes (beliefs) (see “Appendix”) and a semantic differential involving 17 concepts referring to physical and psychosocial aspects of sexuality (each concept^2^ was assessed using 10 pairs of adjectives: good–bad;) to assess the emotional-evaluative aspects of the attitudes.

The discriminatory power ratings of the questions in the scale “The essence of sexuality…” range from 0.28 to 0.70. The scale, consisting of 26 statements, was subject to factor analysis. As a result, three factors were received, all of positive loads, explaining 45.61 % of the results’ variability altogether: the factor of *desexualization and negating the value of sexuality* (32.87 % deviation), the factor of *normalization of sexuality and the complexity of its conditions* (5.77 %) and the factor of *biologization of sexuality* (6.97 %). As a result of the analysis, one question was rejected since it did not belong to any of the factors. The Cronbach’s alpha coefficients for the given factors (factor 1: 0.86, factor 2: 0.83, factor 3: 0.79) and the entire scale (0.92) are satisfactory.

The indicators of discriminatory power ratings for the concepts of the semantic differential range from 0.30 to 0.73. Cronbach’s alpha coefficient for the general result was 0.88.

The number of respondents who participated in the study was 181, with an average age of M = 22.71. They were students of nursing (63 persons), public health (64), emergency medical services (29) and physiotherapy (25). The participants were mostly women (137 persons). Their place of permanent residence varied: 87 persons lived in the city, 91 in smaller towns and villages (no data in 3 cases). Only a few individuals were married (15, of which 10 had children), 77 did not have a permanent partner, while 79 lived in an informal relationship (no data in 10 cases).

## Results

The majority of participants did not have experiences related to the broadly understood sexuality of persons with an intellectual disability. The context of experiences of the respondents who indicated having had them (28 persons) concerned accidental situations (street, park, bus), and less frequently education (13), and concerned mainly to student practices (at social assistance centers, hospitals) or volunteer work. The respondents mentioned various types of behaviors, mainly verbal taunts of a sexual character and exhibitionism, less frequently masturbation. The observed behaviors usually caused their surprise or did not provoke any emotional reactions. The majority of participants (153 persons) did not know any married couples of persons with an intellectual disability or “mixed” couples, i.e., where one of the partners did not have an intellectual disability (149 persons).

The group of respondents who were not taught issues concerning intellectual disability within their curriculum of study (83 persons) was comparable to the group who were taught such issues as an element of a given class (50 persons) or less frequently in a special class devoted to these issues (30 persons). Usually, the content of these classes did not cover issues concerning the sexuality of persons with an intellectual disability.

The participating students most frequently endorsed the social model of intellectual disability (M_INDIVIDUAL_ = 15,45; M_SOCIAL_ = 22,03). A detailed analysis of the statements that this model entails leads to the conclusion that the respondents largely focused on the necessity to normalize the life of these individuals in addition to the need for them to function in their natural environment. They perceived intellectual disability as the result of complex conditions of a biological and psychosocial nature, which simultaneously leads to the conviction that various specialists are needed to cooperate in the process of supporting individuals with such disability. They recognized the civil status of these persons, granting them the opportunity to exercise their rights and fulfill certain duties. The participants were relatively less willing to support the need of these individuals to cooperate in the process of therapy implementation.

In the scope of every notion the respondent could score between 10 and 70 points. A score of 40 points on the differential means a neutral attitude—a given notion is in the middle of the scale with adjectives at each end. Scores lower than 40 mean a negative attitude to a given notion, scores above 40 mean a positive one.

**Graph 1:**
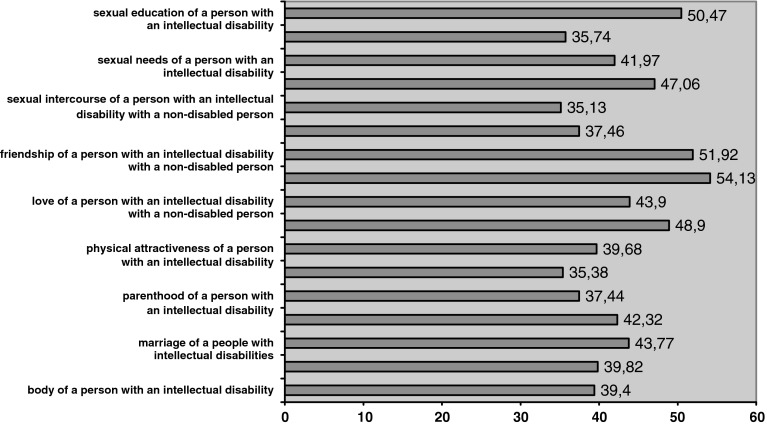
Emotional-evaluative attitudes towards the sexuality of persons with an intellectual disability (semantic differential)

In terms of the emotional-evaluative attitude of students towards the sexuality of persons with an intellectual disability, the highest results were received in the sphere of friendship, both among people with disabilities as well as their relationships with non-disabled individuals (Graph 1). It implies that relationships of this type are the most valued aspect of sexuality of persons with an intellectual disability. Sexual education received lower scores. Positive scores were given to love, mainly to people with an intellectual disability. Lower, but still positive scores, were given to love of a person with an intellectual disability to a non-disabled one. It is worth noting that contraception was assessed relatively high in the hierarchy of concepts. Low scores were given to sexual needs of individuals with an intellectual disability, and even lower to their marital relationships, among which higher scores were received by partnerships of two individuals with disability.

Respondents find it hard to evaluate physical aspects of sexuality such as: physical attractiveness, the sphere of the body, sexual drive or sexual needs of an individuals with intellectual disability; and their responses are rather neutral to these concepts.

Student respondents do not accept fulfilling sexual needs in the form of masturbation, but they expressed the strongest negative attitudes towards sexual intercourse, mainly to relationships between an individual with a disability and a non-disabled one. Although the participants recognized the positive value of contraception in the case of the sexuality of persons with an intellectual disability, they did not accept sterilization as a specific option. Positive assessment of contraception may be related to the lack of acceptance of parenthood among these individuals.

**Graph 2:**
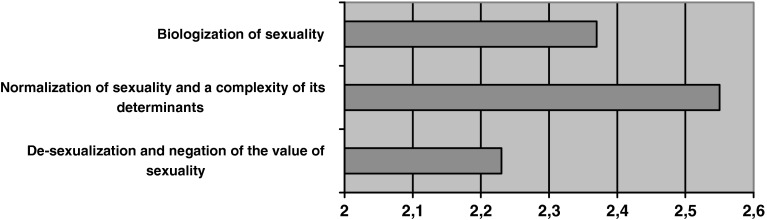
Beliefs about sexuality of persons with an intellectual disability (Likert type scale). (Due to an uneven number of statements in the scales (11, 7 and 7, accordingly) the number of points on the scale was divided by the number of items.)

Beliefs expressed by the students regarding the sexuality of persons with an intellectual disability may mostly be characterized as normalization, although the results obtained here are also similar to those described as desexualization and bipolarization (Graph 2). The normalization approach expressed in the assessments is characterized by beliefs that the factors which condition the sexuality of persons with a disability are complex (in terms of the symptoms of this sexuality and ways of fulfilling it in the form of a marriage), and by their similarity to those factors that are characteristic for the sexuality of non-disabled persons. The respondents also stressed the role of environmental factors (the process of socialization). Another need expressed in the responses was individualization regarding contraception. This attitude was based on beliefs assuming that the determinants and manifestations of sexuality in persons with an intellectual disability are similar to those of non-disabled persons, and they develop towards satisfying the needs of an adult person (not only sexual needs). A characteristic feature of this attitude was the “normalization” of the functioning of a person with disability, especially in its psycho-social and social-sexual dimension.

The bipolarization approach, relatively more weakly expressed, was defined here through certain beliefs regarding the relationship between sexuality and intellectual disability. The respondents were likely to see the intensity of sexual needs in these individuals as deviating from the norm—above all, as much stronger than in non-disabled persons. They expressed beliefs that the sex drive of a person with an intellectual disability is impossible to control as a result of the intellectual deficit, and that it is also a potential threat to the environment due to the lack of control. They attribute persons with an intellectual disability a common practice masturbation. This attitude is, however, not consequent; hence, it should rather be called unbalanced. Nevertheless, a specific feature of this approach is that one stresses a greater sex drive and an inability to control it, although this attitude also comprises beliefs about a weaker intensity of sexual needs in comparison to the norm. The character of statements included in the factor analysis and the figures in the results indicate that this group of beliefs is to be attributed to the biological approach.

The desexualization approach, cohesive in terms of the characteristic opinions, and the least frequently expressed by the respondents, treats persons with a disability as deprived of sexual needs and unable to express their feelings in relationships. A characteristic feature here is that the role of sexual education is perceived as harmful, as it may “awake sexual needs that are dormant”, and sterilization is viewed as a way to protect individuals from sexual abuse.

The correlation analysis indicated a series of significant correlations between the individual model of disability and the emotional-evaluative and cognitive attitude to sexuality (Table 1). The observed relations were in line with the expectations. Hence, the presence of the individual model was accompanied by a negative evaluation of the manifestations sexuality of persons with an intellectual disability, both in its biological, as well as psychosocial dimension. The endorsement of the individual model correlates with a positive valuation of sterilization, and these relations are confirmed in the cognitive dimension of the attitudes. The presence of the individual model is related to two opposing positions: one that assesses the significance of sexuality in the life of persons with a disability as lower than otherwise, as well as one that assesses it as higher than otherwise and tends to perceive it as pathological in its intensity.

**Table 1:** Results of correlation analysis

Scale	Pearson’s correlation coefficients	
	Individual (a)	Social (b)
*Semantic differential*		
(1) Body of a person with an intellectual disability	−0.26*	n.s.
(2) Sex drive of a person with an intellectual disability	−0.21*	n.s.
(3) Marriage of a people with intellectual disabilities	−0.30*	n.s.
(4) Marriage of a person with an intellectual disability with a non-disabled person	−0.24*	n.s.
(5) Parenthood of a person with an intellectual disability	−0.21*	n.s.
(6) Sterilization of a person with an intellectual disability	0.24*	n.s.
(7) Physical attractiveness of a person with an intellectual disability	−0.39*	n.s.
(8) Love of people with intellectual disabilities	−0.34*	0.23*
(9) Love of a person with an intellectual disability with a non-disabled person	−0.39*	n.s.
(10) Friendship of people with intellectual disabilities	−0.16*	0.32*
(11) Friendship of a person with an intellectual disability with a non-disabled person	−0.23*	0.27*
(12) Sexual intercourse of people with intellectual disabilities	−0.44*	n.s.
(13) Sexual intercourse of a person with an intellectual disability with a non-disabled person	n.s.	n.s.
(14) Contraception of a person with an intellectual disability	n.s.	n.s.
(15) Sexual needs of a person with an intellectual disability	−0.34*	n.s.
(16) Masturbation of a person with an intellectual disability	n.s.	n.s.
(17) Sexual education of a person with an intellectual disability	n.s.	0.24*
*The essence of sexuality of persons with an intellectual disability*		
I. De-sexualization and negation of the value of sexuality	0.30*	n.s.
II. Normalization of sexuality and the complexity of its determinants	n.s.	0.29*
III. Biologization of sexuality	0.18*	n.s.

* Significant at *p* < 0.05; *n.s.* non-significant

A few other significant relations of an expected nature have been revealed within the social model. The social model is accompanied by a positive evaluation of relations of love and friendship, as well as sexual education. It is worth mentioning that this correlation was noted only in relation to love between two persons with a disability. The social model is related to an attitude of normalization regarding sexuality, which means that it expresses opinions which let us perceive it as close to the norm in its determinants.

## Discussion

Addressing the initially formulated problems and their hypothetical solutions, we may say that:

1. The majority of the participant students endorsed the social approach to intellectual disability. The expectation based on the conclusions by Scullion [20, 21] and Johnston and Dixon [7]—that due to the specific character of the content of their education, the respondents would be likely to perceive intellectual disability in the categories referring to the individual (medical) model—was not confirmed. It might be that their attitude to this issue is an expression of certain socio-cultural changes taking place in relation to treating disability across generations [22]. Such an interpretation is especially likely to be true, as the college curriculum of only half of the students included contents related to intellectual disability; hence, it may be expected that beliefs regarding disability expressed in the survey have their roots elsewhere. However, to verifying the hypothesis of a change of attitudes across generations would require the analysis of attitudes towards various types of disabilities and the inclusion of the age factor.

It can be expected that the expressed attitudes will be reflected in the professional practice of the participating students. However, undoubtedly, to undertake activities that are characteristic for the social model of disability (e.g., equal treatment in terms of the access to rights, co-operation in the support process) it is necessary to meet certain legal-organizational conditions that depend on these specialists only to a limited degree.

2. The emotional-evaluative attitude is relatively the most positive (highest) when considering friendly relationships between persons with an intellectual disability and non-disabled persons as well as sexual education. Taking into consideration the remaining results of evaluations it seems that the image of the desired sexuality of persons with a disability expressed by the respondents has the qualities of platonic love. Highly valued relationships were based on friendship and love (to a lesser degree), but lacked the aspects of physical sexuality. Fulfilling sexual needs by individuals with an intellectual disability both in the form of sexual intercourse and masturbation was not accepted by the respondents. Low scores were also given to some physical aspects of these individuals’ sexuality such as needs and sexual drive. Such evaluations are revealed by the emotions and feelings assigned to certain concepts; hence, it may not be excluded that the physical sphere of intellectually disabled persons raises more negative emotions than the sphere of their emotional and social experiences. Similar tendencies in terms of evaluations were found in studies on students of pedagogy [23] and on specialists working with persons with disabilities [2, 24]. Also, convergent results were obtained during the study with hospital personnel respondents. Among others, liberal attitudes were found towards emotional relations of persons with an intellectual disability in heterosexual relationships but lack of consent to sexual intercourse, and conversely to the study by Yool [25], liberal attitudes towards masturbation. Other studies with support staff showed lack of acceptance towards sexual behaviors of individuals with an intellectual disability [26]. On the basis of studies with staff carers and family carers Evans et al. [24] found that the most desirable are friendly and non-intimate relationships between an individuals with intellectual disability. The variable which differentiated opinions about it was the severity of intellectual disability. Study results varied if they included intellectual abilities of partners (person with intellectual disability—non-disabled person). Author’s research suggests that homogenous relationships (persons with an intellectual disability) are more acceptable. This hypothesis was partially corroborated by studies with pedagogues working with persons with an intellectual disability and Pedagogy students [23, 27], however other studies with various specialists proved otherwise [18]. Morales et al. [28] showed in studies with parents and guardians that acceptance of sexual relations depends on various factors, among others: sex, level of autonomy, contraception methods used and the age of partners. A significant differentiating factor in determining attitudes towards sexual relationships of individuals with intellectual disability is the issue of them being able to consciously give consent [29].

The issue worth considering is sterilization which raises a lot of controversy and is not accepted by the respondents. Research carried out to date devoted to this type of control over sexuality of individuals with disabilities suggests divergence in evaluation and opinions, which is related to the variety of the adopted criteria for attitudes [18, 23, 30–32].

What may be puzzling here, is the negative evaluation of masturbation which in cases of individuals with a more severe intellectual disability is the only available form of fulfilling one’s sexual drive [33, 34]. Evidence in favor of this form, but conditional ones, can be found in literature, where it is seen as a more favorable form than sexual intercourse [25, 30]. However, there is evidence for negative attitudes to masturbation, very often connected with stereotypical way of thinking about it and incidents of performing it in public places [35, 36].

The beliefs expressed by the respondents regarding the sexuality of persons with an intellectual disability varied. A slightly stronger represented attitude was one of normalization, stressing that the sexuality of disabled persons is conditioned by a variety of determinants; however, a similarly represented attitude concerned unfavorable attitudes of desexualization and biologization. Most of the participants mentioned that in the course of their education they did not encounter issues referring to the sexuality of persons with an intellectual disability. This might have been the decisive factor due to which the expressed beliefs on the sexuality of persons with an intellectual disability were so varied. It is likely that for the majority of respondents the source of such beliefs were the views and beliefs of others, including persons who were important for them (parents, friends, relations); in certain cases it might have been literature, while—according to the results—personal experiences were rare. The lack of formal, organized education that would allow them to gain reliable and relatively comprehensive knowledge should be treated as a factor that is potentially harmful for professional functioning of the participants in the future [24, 37]. Results obtained here should be interpreted in relation to the affective-evaluative aspect—this may help to explain the inconsistencies of attitudes found in the research. Students perceive individuals with an intellectual disability as sexual beings, yet, as aforementioned, they accept certain aspects of this sexuality to various degrees. Some of these aspects are more easily accepted, others to a lesser degree and still others not accepted at all. Among the last group are especially these that have social consequences in relationships—sexual intercourse and procreation. Studies have shown that specialists give positive scores to sexuality of individuals with an intellectual disability and treat it as normal and natural, emphasizing the need of sexual education, but also display ambivalence or negative attitudes towards procreation of such individuals. Morales et al. [28] observe that “overall, attitudes towards the sexuality of people with an intellectual disability are moderately positive, more liberal now than they were 20 years ago (…) respondents’ main concerns, presently, are more centered on the consequences of the sexual relationships than on the relationships per se”. One of such consequences is parenthood. Numerous studies with parents, guardians and specialists have proved it to be the least accepted, compared to others, aspect of sexuality [38] which is also reflected in various convictions about the nature of intellectual disability [39–41].

3. The observed relationships between the individual and social models and attitudes towards the sexuality of persons with an intellectual disability are in line with the expected. Perceiving intellectual disability as a biological, irrevocable phenomenon (as a result of damage to the central nervous system), also suggesting limited conditions of life and the inability to decide about oneself, is related to the low valuation of the sexuality of persons with this disability, in its biological and psychosocial dimension, and leads to accepting sterilization as a method of blocking fertility permanently. It also co-occurs with negative beliefs which suggest that disabled persons are asexual or that sexuality is biologically determined. The social model co-occurs with a positive valuation of the sexuality of people with disabilities in its psychosocial and educational dimension, as well as with opinions that assume that its determinants are complex and in line with the determinants of the sexuality of non-disabled persons. One may thus conclude that the sexuality of persons with an intellectual disability is an element of their social assessment, and of the disability as such, also related to a certain perception of the types of support offered.

## Conclusion

The analysis of the presented issue needs further elaboration. Due to the limited length of the paper and the specific distribution of the variables, the study omitted the potentially differentiating socio-demographic aspects (age, sex, place of permanent residence, family status), as well as the specific field of studies. Of special significance here seems the age variable, although among the population of students in the present study it is of limited relevance (the narrow age range limits the possibility of analyzing transformations among the generations). In the context of the relevant studies to date, it seems justified to consider the respondents’ personality traits, such as empathy or the system of values, as well as their general attitudes towards persons with a disability.

To explain certain issues, such as how the endorsed model is actually expressed in reality, or how certain attitudes are revealed, would require a two-stage study, conducted first at college, and then when the graduates are already active professionally. Although for various reasons this postulate is difficult to satisfy, certain value may be expected from surveys carried out on students from different years of studies or at different stages of their professional experience.

## Appendix

### Items in Individual Model Scale

An individual with intellectual disability should function in a closed environment (an institution).

Individuals with intellectual disability are bound to be reliant on others.

Intellectual disability results from CNS damage.

Individuals with intellectual disability are not able to take decisions on issues related to their own rehabilitation.

Individuals with intellectual disability are not able to enjoy civil rights and duties.

Intellectual disability requires mainly medical intervention.

### Items in Social Model Scale

Intellectual disability results from complex biological, psychological and social factors.

Support for individuals with intellectual disability requires cooperation between a number of specialists.

An individual with intellectual disability should be given natural open living conditions.

As far as possible individuals with intellectual disability should be included in the process of taking decisions on issues related to their own support.

With proper preparations and support individuals with intellectual disability are able to function more independently.

Individuals with intellectual disability are citizens as we all are and as far as possible should be able to enjoy civil rights and duties.

### Scale “The Essence of Sexuality of Persons with an Intellectual Disability”

#### Desexualization and Negating the Value of Sexuality

Intellectual disability eliminates sexual needs.

Individuals with intellectual disability are in most cases “forever children” and require constant care.

Individuals with intellectual disability are in most cases incapable of forming marital relationships.

Individuals with intellectual disability usually express their sexual needs in pathological forms.

Due to deficits in the cognitive and the emotional sphere individuals with intellectual disability are usually incapable of fidelity in a partnership.

Intellectual disability is always inherited, hence individuals with disability should not be allowed to have children.

Love in a partnership of individuals with intellectual disability is in most cases only an attempt to copy observed models and not a genuine feeling.

Sterilization would protect individuals with intellectual disability from sexual harassment.

Individuals with intellectual disability tend to commit sexual-related crimes.

Bringing up issues related to sexuality e.g., during school education may “arouse dormant sexual needs”.

Individuals with intellectual disability are very unlikely to form happy relationships with each other.

#### Normalization of Sexuality and the Complexity of its Conditions

Sexual needs of individuals with intellectual disability may be socialized just as the needs of non-disabled individuals.

Masturbation of individuals with intellectual disability may be emotionally conditioned.

Intellectual disability does not eliminate the possibility of developing needs related to adulthood such as procreation and the need for intimate relationship.

The quality of sexuality of individuals with intellectual disability is often shaped by the same factors which shape sexuality of non-disabled individuals.

Success of a marital relationship of individuals with intellectual disability often depends on the same factors which determine the success of a marital relationship of non-disabled individuals.

It would be most appropriate if contraception used by individuals with intellectual disability suited their individual needs.

Masturbation in public places often results from negligence and mistakes made in the process of child-rearing.

#### Biologization of Sexuality

Sexual needs in individuals with intellectual disability are often stronger than in non-disabled individuals.

Sexual needs in individuals with intellectual disability are often weaker than in non-disabled individuals.

Individuals with intellectual disability are not able to control their sexual drive.

The strength of sexual drive in individuals with intellectual disability is often related to their intelligence.

Most individuals with intellectual disability masturbate.

Uncontrolled sexuality of individuals with intellectual disability poses a serious threat to society.

Individuals with intellectual disability who seek heterosexual relationships often want to fulfill their mental and physical needs.

## Footnotes

1 Activities that are specific for the given model of disability concerning the sexuality of persons with a disability should be analyzed in the context of the western culture, where therapy and support for them were most advanced.

2 Concepts have been mentioned in analysis of results (Graph 1).

## References

[1] Kirenko J: Indywidualna i społeczna percepcja niepełnosprawności (Individual and social perception of disability). UMCS, Lublin (2007)

[2] Chivers J, Mathieson S (2000). Training sexuality and relationships: an Australian model. Sex. Disabil..

[3] Anastasiou D, Kauffman J (2011). A social constructionist approach to disability: implications for special education. Except. Children..

[4] Terzi L (2004). The social model of disability: a philosophical critique. J. Appl. Phil..

[5] Shakespeare T (2000). Disabled sexuality: toward rights and recognition. Sex. Disabil..

[6] Rembis MA (2010). Beyond the binary: rethinking the social model of disabled sexuality. Sex. Disabil..

[7] Johnston C., Dixon R.: Nursing students’ attitudes towards people with disabilities: can they be changed? www.aare.edu.au/98pap/joh98196.htm

[8] Tervo RC, Palmer G, Redinius P (2004). Health professional student attitudes towards people with disability. Clin. Rehabil..

[9] Sahin H, Akyol AD (2010). Evaluation of nursing and medical students’ attitudes towards people with disabilities. J. Clin. Nurs..

[10] Aulagnier M, Verger P, Ravaud J-F (2005). General practitioners’ attitudes towards patients with disabilities: the need for training and support. Disabil. Rehabil..

[11] Tracy J, Graves P (1996). Medical students and people with disabilities: a teaching unit for medical students exploring the impact of disability on the individual and the family. Med. Teach..

[12] Tracy J, Iacono T (2008). People with developmental disabilities teaching medical students—does it make a difference?. J. Intellect. Dev. Disabil..

[13] Dąbkowska M., Błeszyński J., Dąbkowski M.: Postawy studentów pedagogiki oraz medycyny wobec dzieci z upośledzeniem umysłowym (Attitudes of students of pedagogy and medicine towards children with intellectual disability). In: Kosakowski, Cz., Krause, A., Wójcik, M. (eds.) Relacje i doświadczenia społeczne osób z niepełnosprawnością (Social rates and experiences of individuals with disability), pp. 200–206. AKAPIT, Toruń (2009)

[14] Klooster PM, Dannenberg J-W, Taal E (2009). Attitudes towards people with physical or intellectual disabilities: nursing students and non-nursing peers. J. Adv. Nurs..

[15] Jones L, Binger T, McKenzie C (2010). Sexuality, pregnancy and midwifery care for women with intellectual disabilities: a pilot study on attitudes of university students. Contemp. Nurse.

[16] Grieve A, McLaren S, Lindsay W, Culling E (2008). Staff attitudes towards the sexuality of people with intellectual disabilities: a comparison of different professional groups and residential facilities. Br. J. Learn. Disabil..

[17] Swango-Wilson A (2009). Perception of sex education for individuals with developmental and cognitive disability: a four cohort study. Sex. Disabil..

[18] Parchomiuk M.: Specialists and sexuality of individuals with disability. Sex Disabil. (Online first). doi:10.1007/s11195-011-9249-x

[19] McCarthy M (2011). Prescribing contraception to women with intellectual disabilities: general practitioners’ attitudes and practices. Sex. Disabil..

[20] Scullion PA (2010). Models of disability: their influence in nursing and potential role in challenging discrimination. J. Adv. Nurs..

[21] Scullion P (1999). Conceptualizing disability in nursing: some evidence from students and their teachers. J. Adv. Nurs..

[22] Kazanowski Z.: Przemiany pokoleniowe postaw wobec osób upośledzonych umysłowo (Generational transformations of attitudes towards intellectually disabled persons). UMCS, Lublin (2011)

[23] Parchomiuk, M.: Postawy studentów pedagogiki wobec życia erotycznego osób niepełnosprawnych (Pedagogy student’s attitudes towards sex life of disabled persons). Człow. Niepełnospr. Społecz. **1**, 93–105 (2007)

[24] Evans D, McGuire B, Healy E, Carley S (2009). Sexuality and personal relationships for people with an intellectual disability. Part II: staff and family career perspectives. J. Intellect. Disabil. Res..

[25] Yool L, Langdon P, Garner K (2003). The attitudes of medium—secure unit staff towards the sexuality of adults with learning disabilities. Sex. Disabil..

[26] Trudel G, Desjardins G (1992). Staff reactions toward the sexual behaviors of people living in institutional settings. Sex. Disabil..

[27] Parchomiuk M.: Postawy pedagogów i studentów pedagogiki wobec seksualności osób z niepełnosprawnością intelektualną (Pedagogues and pedagogy student’s attitudes towards sexuality of persons with an intellectual disability). Manuscript

[28] Morales G, Ramirez Lopez E, Mullet E (2011). Acceptability of sexual relationships among people with learning disabilities: family and professional caregivers’ views in Mexico. Sex. Disabil..

[29] Fader Wilkenfeld B.: Educators’ attitudes and beliefs towards the sexuality of individuals with developmental disabilities. Sex Disabil. **29**, 351–361 (2011)

[30] Wolfe P (1997). The influence of personal values on issues of sexuality and disability. Sex. Disabil..

[31] Christian LA, Stinson J, Dotson LA (2001). Staff values regarding the sexual expression of women with developmental disabilities. Sex. Disabil..

[32] Parchomiuk M.: Seksualność osób z upośledzeniem umysłowym w opinii rodziców i studentów pedagogiki (Sexuality of persons with an intellectual disability in opinion of parents and students of pedagogy). Lub. Rocz. Pedagog. **18**, 39–54 (2009)

[33] Kijak R.: Seks i niepełnosprawność. Doświadczenia seksualne osób z niepełnosprawnością intelektualną (Sex and disability: the sexual experience of people with an intellectual disability). Impuls Krak. (2009)

[34] Gomez Taylor M: The S words: sexuality, sensuality, sexual expression and people with intellectual disability. Sex Disabil. **30**, 237–245 (2012)

[35] Hogg J, Campbell M, Cullen C, Hudson W (2001). Evaluation of the effect of an open learning course on staff knowledge and attitudes towards the sexual abuse if adult s with learning disabilities. J. Appl. Res. Intellect. Disabil..

[36] Lockhart K, Guerin S, Shanahan S, Coyle K (2009). Defining “sexualized challenging behavior” in adults with intellectual disability. J. Policy Pract. Intellect. Disabil..

[37] Abbott D, Howarth J (2007). Still off-limits? Staff views on supporting gay, lesbian and bisexual people with intellectual disabilities to develop sexual and intimate relationships?. J. Appl. Res. Intellect. Disabil..

[38] Cuskelly M, Bryde R (2004). Attitudes towards the sexuality of adults with an intellectual disability: parents, support staff, and a community sample. J. Intellect. Dev. Disabil..

[39] McCarthy M (2009). ‘I’have the jab so I can’t be blamed for getting pregnant’: contraception and women with learning disabilities. Womens Stud. Int. Forum.

[40] Aunos M, Feldman M (2002). Attitudes towards sexuality, sterilization and parenting rights of persons with intellectual disabilities. J. Appl. Res. Intellect. Disabil..

[41] Areschoug J (2005). Parenthood and intellectual disability: discourses on birth control and parents with intellectual disabilities 1967–2003. Scand. J. Disabil. Res..

